# Impact of Sleep Telementorship in Primary Care: Sleep VA-ECHO (Veterans Affairs-Extension for Community Healthcare Outcomes)

**DOI:** 10.3390/ijerph18189914

**Published:** 2021-09-21

**Authors:** Brian N. Palen, Elizabeth A. Mattox, Ken He, Lauren A. Beste, Joleen Borgerding, Sarah Patel, David H. Au, Michael F. Chang, Elizabeth C. Parsons

**Affiliations:** 1 Pulmonary and Critical Care Section, Veterans Affairs Puget Sound Health Care System, Seattle, WA 98108, USA; ken.he@va.gov (K.H.); david.au@va.gov (D.H.A.); elizabeth.parsons@va.gov (E.C.P.); 2 Division of Pulmonary, Critical Care, and Sleep Medicine, University of Washington, Seattle, WA 98195, USA; 3 General Medicine Service, Veterans Affairs Puget Sound Health Care System, Seattle, WA 98108, USA; lauren.beste@va.gov; 4 Health Services Research and Development Center for Innovation, Veterans Affairs Puget Sound Health Care System, Seattle, WA 98108, USA; joleen.borgerding@va.gov; 5 Department of Medicine, University of Arizona, Tucson, AZ 85721, USA; sarah.patel@gmail.com; 6 Sonoran Sleep Center, Glendale, AZ 85306, USA; 7 Gastroenterology and Hepatology Service, Veterans Affairs Portland Health Care System, Portland, OR 97239, USA; Michael.Chang2@va.gov; 8 Gastroenterology and Hepatology Division, Oregon Health & Sciences University, Portland, OR 97239, USA

**Keywords:** sleep disorders, primary care, education, mentorship, health professions education, professional development, virtual education, project ECHO

## Abstract

Sleep VA-ECHO (Veterans Affairs–Extension for Community Healthcare Outcomes) is a national telementorship program intended to improve knowledge about sleep disorders among non-specialty providers. The project goal was to describe the characteristics of Sleep VA-ECHO participants from primary care and their use of program-obtained knowledge in practice. Sleep VA-ECHO consisted of 10 voluntary, 75-min teleconference sessions combining didactics and case discussion. Out of 86 participants, 21 self-identified as primary care team members and completed a program evaluation. Participants self-reported their application of knowledge gained, including changes to practice as a result of program participation. These 21 participants represented 18 sites in 11 states and attended a median of 5.0 sessions. They included physicians (29%), nurse practitioners (24%), and registered nurses (24%). Nearly all participants (95%) reported using acquired knowledge to care for their own patients at least once a month; 67% shared knowledge with colleagues at least once a month. Eighty-five percent reported improved quality of sleep care for their patients, and 76% reported an expanded clinical skillset. The greatest self-reported change in practice occurred in patient education about sleep disorders (95%) and non-pharmacologic management of insomnia (81%).

## 1. Introduction

Sleep disorders are highly prevalent, yet underdiagnosed, and often undertreated [1,2,3]. A national shortage of sleep specialists in the United States (US) [1,4] and their concentration in urban centers [4,5] further contributes to these deficiencies by shifting the burden of sleep care to primary care providers. Addressing these and other deficits in sleep care is a major focus for the United States (US) Department of Veterans Affairs (VA) Veterans Health Administration (VHA) [6,7].

Primary care encounters represent pivotal opportunities for screening and care coordination for common sleep disorders such as obstructive sleep apnea (OSA) and insomnia [4,8,9,10,11] Unfortunately, most primary care providers receive little or no formal education in sleep medicine limiting their delivery of sleep care [1,12,13]. Creating a community of practice that engages primary care teams in basic sleep education, with the intent to extend specialty sleep care, is one proposition to bridge the sleep care gap. In 2015, we developed a national sleep medicine telementorship program for VA healthcare providers called Sleep VA-ECHO (Veterans Affairs–Extension for Community Healthcare Outcomes). Our program uses the Project ECHO (Extension for Community Healthcare Outcomes) framework (described below), which combines live online educational content presented by specialists with the real-time clinical case review. After participating in our 2015 Sleep VA-ECHO pilot program, most participants self-reported increased knowledge about common sleep disorders like sleep apnea and insomnia [14].

Project ECHO, developed by Dr. Sanjeev Arora in 2003 at the University of New Mexico (UNM), is a strategy for improving healthcare delivery to underserved populations through the creation of knowledge networks composed of specialty care providers and primary team members without specialty training. In 2011, the UNM-based team reported that rural and underserved patients with hepatitis C served through Project ECHO had similar outcomes when compared to those receiving care at tertiary healthcare settings [15,16].

The UNM experience prompted the implementation of Project ECHO and ECHO-like models (EELMs) throughout the world, focusing on a wide range of health conditions, as well as education and civics [17]. The VA initially established seven VA-ECHO hubs, focusing on a variety of disease states, including chronic renal failure, pulmonary nodules, cancer, and pain management in addition to hepatitis C. Several programs expanded their focus over time. Sleep VA-ECHO emerged from Pulmonary VA-ECHO in response to significant interest among participants in additional and more regular sleep content. At the time of implementation, our program was one of the only (if not the only) EELM focused on adult sleep medicine.

Despite the initial promise of Project ECHO, objective evaluation of benefit, including provider performance, patient health and population health, has remained limited [16,18,19]. Most published evaluations focus on subjective outcomes such as participant reports of self-efficacy. While these consistently demonstrate subjective improvement in self-efficacy, almost all evaluations face a similar combination of limitations—small sample size, disparate application of the Project ECHO framework, low response rates, and response bias [18]. Some theorize that the initial outcomes reported in patients with hepatitis C are not necessarily generalizable to other conditions [16].

While our pilot program was shown to improve self-reported knowledge of sleep care among participants, it is unknown how or if that translates to changes in practice and performance. We present our program’s second-year evaluation, describing specifically the primary care team members (PCTMs) who participated, ways in which they applied knowledge gained, and self-reported changes in their clinical practice within specific domains of sleep medicine.

## 2. Materials and Methods

VHA is the geographically largest integrated healthcare system in the US. VHA Northwest Health Network (VISN 20), is one of 18 regional VA health networks and includes 6 medical centers and 46 additional sites of care in Washington, Oregon, Idaho, Alaska, northern California and western Montana [20]. Our Sleep VA-ECHO program was created in 2015 to improve sleep care within VISN 20 and is led by sleep medicine specialists at VA Puget Sound in Washington State.

Sleep VA-ECHO participation was voluntary and not linked to compensation or administrative incentives. Participants were recruited through emailed program announcements, first sent in June 2016 to select regional clinical leaders, preexisting VISN 20 VA-ECHO email lists, and relevant national program email lists. We encouraged recipients to share the announcements with colleagues. Over 1800 individuals received the announcements; 224 potential participants completed an online enrollment form to receive session invitations.

The general structure of Sleep VA-ECHO has been described in detail [14]. The 2016 program offered ten 75-min stand-alone sessions on basic sleep medicine topics conducted weekly between 15 September and 17 November 2016. Video teleconferencing connected program faculty with participants from multiple clinical locations. Specialists from sleep medicine and mental health led didactic sessions integrated with up to two interactive case reviews. The content was based on the results of prior needs assessments, with the greatest focus on OSA and insomnia. Cases were selected from referrals to VA Puget Sound Sleep Medicine.

Attendance tracking methods and logistics are described previously [14]. Participant characteristics were collected during initial enrollment and at final program evaluation, including credentials, practice setting (urban vs. rural), self-reported prior sleep medicine training, and frequency of sleep complaints in their practice.

Participants received a final online program evaluation 4 weeks after the final session using Research Electronic Data Capture (REDCap). The response was voluntary but identifiable (required for continuing education credit). Participants were offered a sleep medicine reference guide with a nominal value of USD 10 to complete the evaluation.

The final evaluation included Likert-type scales asking participants to report program impact on the following: “How has your comfort level with specific aspects of clinical practice changed?” and “To what extent did your clinical practice change?”. Additional multiple-choice questions asked participants to indicate specific ways in which they used knowledge gained, including care of their own patients and helping colleagues.

We generated descriptive statistics for participant demographics and survey responses on subjective changes in comfort and clinical practice related to sleep disorders. We also present descriptive statistics on multiple-choice survey items in which participants were asked to indicate specific ways in which they used Sleep VA-ECHO knowledge gained.

This approved quality improvement initiative was conducted under the auspices of the VA Office of Specialty Care and the Office of Rural Health. In accordance with VHA Handbook 1058.05, we obtained approval of non-research status from the VA program office. In addition, we secured written concurrence of non-research status from VA Puget Sound’s Director of Human Research Protection Program, Associate Chief of Staff for Research and Development, and Director of Quality, Safety and Values. As a designated quality improvement (non-research) project, informed consent was not obtained.

## 3. Result

Of 86 multidisciplinary participants in 2016 Sleep VA-ECHO, 30 self-identified as PCTMs. Twenty-one completed the final program evaluation (70% response rate). Responding participant characteristics are detailed in Table 1. Participants attended a median of 5.0 sessions (IQR 4.0). The most represented disciplines were physicians (MDs) (29%), advanced registered nurse practitioners (ARNPs) (24%), and non-advanced practice registered nurses (RNs) (24%). The remaining 23% were comprised of the following: healthcare trainee psychologist, licensed practical nurse, Doctor of Philosophy (Ph.D.) trained mental health provider, psychologist (PsyD), and Doctor of Pharmacy (PharmD).

Nineteen PCTMs responded to questions about their clinical practice. They worked in primary care for a median of 8.0 years (range 6 months–25 years). Most (71%) reported very limited or no prior sleep medicine education, and 55% reported that sleep services for their patients were limited and required patient travel.

**Figure 1:**
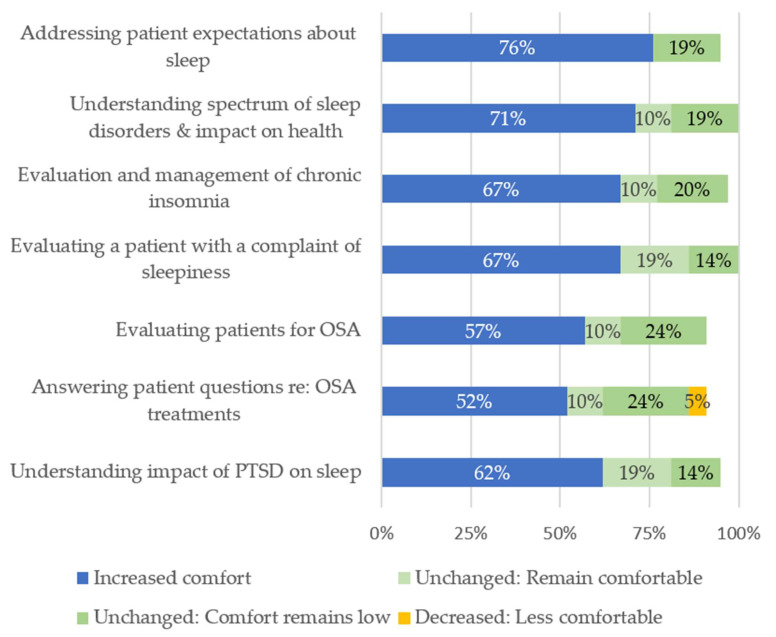
Self-reported change in comfort managing specific aspects of sleep care (n = 21). Abbreviations: OSA: obstructive sleep apnea; PSTD: post-traumatic stress disorder. Denominators include “not applicable” and “do not know” responses, but these categories are not displayed.

Most respondents reported increased comfort managing aspects of sleep care covered in the program (Figure 1). Respondents reported the greatest increase in comfort educating patients about realistic expectations of their sleep (76%), a topic covered during multiple sessions and case discussions. Respondents reported the smallest increase in comfort for OSA evaluation (57%) and answering patient questions about OSA treatment (52%). A single ARNP reported decreased comfort in this last area.

**Figure 2:**
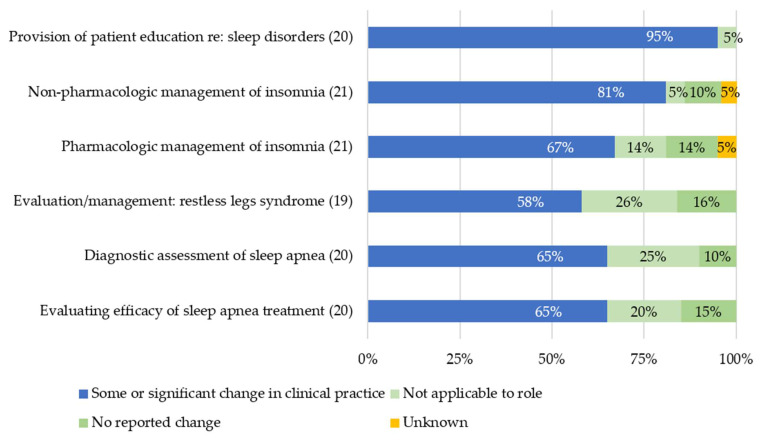
Self-reported change in clinical practice. Denominators, noted after each domain, exclude those who answered “did not attend a session related to this content”.

Most PCTMs reported changing their clinical practice as a result of participation (Figure 2). The greatest practice change was in patient education about sleep disorders (95%) while behavioral management of change related to OSA diagnosis and management was considerably lower (65%). Some PCTMs reported that diagnostic evaluation of OSA and/or evaluation of OSA treatment efficacy were not applicable to their role; these included two ARNPs, a PsyD, an MD, and a PharmD.

**Figure 3:**
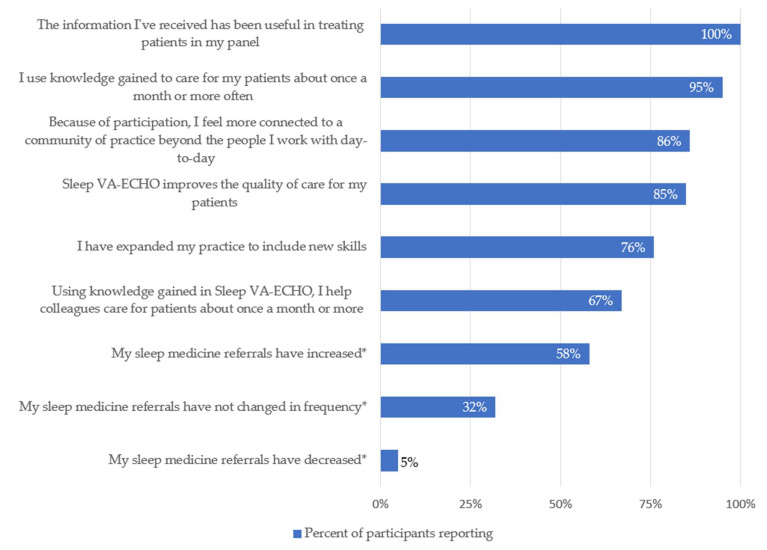
Specific ways participants report using knowledge gained. Abbreviations: VA-ECHO: Veterans Affairs–Extension for Community Healthcare Outcomes; * mutually exclusive options.

PCTMs report frequently using the knowledge gained through participation: 52% reported utilization about once a month, 24% reported about once a week, and 19% reported utilization more than weekly. PCTMs also reported multiple ways in which Sleep VA-ECHO participation impacted their clinical practice. Fifty-eight percent reported an increased frequency of referrals to sleep medicine. Eighty-five percent reported improved quality of sleep care for their patients as a result of participation, and 76% reported an expanded clinical skillset (Figure 3).

Sleep VA-ECHO participation increased the sense of community of practice among most participating PCTMs (86%). Sixty-seven percent of respondents reported using VA-ECHO knowledge to help colleagues about once a month or more often; this group included MDs, ARNPs, and RNs.

## 4. Discussion

Participants from primary care attending our Sleep VA-ECHO program included multiple members of the comprehensive primary care team including nurses, advanced registered nurse practitioners, and physicians. These participants consistently reported = change in their clinical practice related to patient education about sleep disorders and behavioral management of insomnia, with lesser impact to clinical practice related to OSA care. Additional benefits noted by participants included a perception of higher quality of sleep care for their patients, an expansion of personal clinical skillset, and regular dissemination of new knowledge to both patients and colleagues (force multiplication)

The objective impact of Sleep VA-ECHO on provider performance and patient-level outcomes has not yet been determined. Our analysis suggests that knowledge gains did not universally translate to increased self-efficacy in sleep disorder management or to changes in clinical practice. These disparate findings are consistent with other EELMs and highlight the challenge in quantifying the clinical impact of telementorship in a broad and multidisciplinary specialty like sleep medicine. Our results suggest that PCTMs consider patient education and management of insomnia within their scope of practice but may not have the same perception about the management and evaluation of OSA. Existing Sleep VA-ECHO program structure and care metrics likely need to be tailored to these and other specific provider needs, interests, and practice scope. In content areas like OSA, program success may hinge not just on education, but on facilitating provider and patient access to on-demand resources such as straightforward algorithms for OSA screening, testing, and treatment along with patient-driven monitoring and appropriate, patient-centered feedback that is actionable and enhances self-reliance.

A majority of participants (86%) reported that Sleep VA-ECHO made them feel more connected to a community of practice beyond the people they work with day-to-day. Building a sense of community may reduce provider burnout and improve workforce retention in geographically isolated sites [21], as well as impact referral patterns within healthcare systems. EELMs have the potential to facilitate peer-to-peer learning and support, much of which has been lost given the persistent demands on providers [22], a loss even more pronounced during the COVID-19 pandemic. The concept that EELMs promote trust and improved understanding among healthcare team members is important, particularly with respect to the impact on healthcare delivery. Dr. Max Watson, director of the Project ECHO at Hospice UK contends that EELMs change the way team members work together and creates an environment for solutions to emerge [23]. Moving forward, EELM implementors would be best served to monitor the health and trust of this community of practice [23], as well as investigating evaluation strategies that consider the impact of new communities of practice.

An under-recognized part of creating a successful community of practice is engaging all team members, not just physicians. Advanced registered nurse practitioners and RNs were highly represented among our PCTM participants. RNs, in particular, may represent an underutilized resource for sleep care outreach–with potential roles in patient education about sleep hygiene, behavioral interventions for insomnia, and anticipatory guidance in advance of sleep testing. Creating specific telementorship for RNs, enhancing their clinical scope, and focusing on high-demand and algorithmic interventions, may help extend sleep specialty services without requiring additional independent practitioners.

Another key element of the ECHO community of practice is “force multiplication” in which participants carry knowledge forward and become teachers. We were pleased to see that 67% of our participants shared knowledge gained with colleagues on a monthly or more basis.

Our project has several limitations, many of which are common among EELM evaluations. Our sample size of PCTM participants was small and may not represent non-VA providers. Responder bias was possible as both participation and evaluations were voluntary and identifiable, and individuals who disliked the program may have declined to return evaluations. Self-reported changes in practice are inherently subjective and may not reflect objective changes in practice or referral patterns, which should be evaluated in future work. Sleep VA-ECHO may increase the visibility of and demand for specialty services at the program facility (58% of our program respondents reported an increase in sleep referrals) that could lead to further strain on limited resources. This program likely has a complex impact on sleep care delivery that should be evaluated further, with an attempt to discern the impact of a community of practice development as well as knowledge gains.

Several important questions remain about how to optimally implement sleep care outreach into the primary care setting. Primary care teams are already overburdened with high patient volume, time constraints, and limited infrastructure. Teams developing outreach programs need to explicitly acknowledge these pre-existing limitations. Our experience suggests that sleep education tailored to the facets of care that PCTMs consider within their scope of practice (e.g., insomnia) will have the greatest impact on patient care and outcomes. Certain aspects of sleep care, including complex OSA management, will likely remain beyond the feasible scope for most PCTMs and have less value in an outreach curriculum. Content and case-based learning that does not reflect participant scope of practice risks wasting valuable resources (time and energy) and eroding trust between ECHO teams and participating providers. Ultimately, a comprehensive sleep care outreach program needs to be multifaceted–combining tailored education, traditional telehealth, and interdisciplinary care teams that deploy sleep specialty resources and support staff to remote sites.

## 5. Conclusions

PCTMs report numerous benefits from Sleep VA-ECHO, including improved quality of care for their patients, an expanded skillset in managing sleep issues in their patients, and an increased sense of community. They reported the greatest benefit to their clinical practice in patient education and behavioral management of insomnia, and a lesser benefit in OSA care. Evaluation of the impact of sense of community among providers on care delivery may inform the objective evaluation of provider performance, and patient and population outcomes of EELMs and sleep outreach evaluation. Sleep telementorship may impact patients the most as part of a multifaceted intervention that includes education tailored to provider needs, and deployment of specialty care resources to remote sites.

## Figures and Tables

**Table 1:** Sleep VA-ECHO Primary Care Team Member Characteristics, N = 21.

Participant Characteristics	
sessions attended, median (IQR)	5.0 (4.0)
credentials, n (%)	
physician (MDs)	6 (29%)
advanced registered nurse practitioners (ARNPs)	5 (24%)
registered nurses (RNs)	5 (24%)
other	5 (23%)
duration working in primary care, median (IQR)	8.0 (10.0) years
prior exposure to sleep medicine, n (%)	
none to very limited	15 (71%)
limited	2 (10%)
moderate	4 (19%)
extensive	0 (0%)
percentage of patients with sleep complaints evaluated or managed in a typical clinical day, n (%)	
1–14%	9 (43%)
15–49%	8 (38%)
50–75%	3 (14%)
76–100%	1 (5%)
Practice Setting	
rural, n (%)	5 (24%)
sleep service availability, n (%) ^†^	
limited, requires patient travel	6 (55%)
limited but locally available	2 (18%)
readily available	3 (27%)

^†^ 11 respondents.

## Data Availability

Data available on request from corresponding author. The data are not publicly available due to inclusion of comments which may render respondents identifiable, particularly in the context of low sample size.
